# Metabolite Biomarkers for Early Ischemic–Hypoxic Encephalopathy: An Experimental Study Using the NeoBase 2 MSMS Kit in a Rat Model

**DOI:** 10.3390/ijms25042035

**Published:** 2024-02-07

**Authors:** Yulia Shevtsova, Natalia Starodubtseva, Alisa Tokareva, Kirill Goryunov, Alsu Sadekova, Irina Vedikhina, Tatiana Ivanetz, Oleg Ionov, Vladimir Frankevich, Egor Plotnikov, Gennady Sukhikh, Dmitry Zorov, Denis Silachev

**Affiliations:** 1 V.I. Kulakov National Medical Research Center for Obstetrics Gynecology and Perinatology, Ministry of Healthcare of Russian Federation, 117997 Moscow, Russia; yu_shevtsova@oparina4.ru (Y.S.); n_starodubtseva@oparina4.ru (N.S.); a_tokareva@oparina4.ru (A.T.); k_gorunov@oparina4.ru (K.G.); sadekovaaa4@gmail.com (A.S.); i_vedikhina@oparina4.ru (I.V.); t_ivanets@oparina4.ru (T.I.); o_ionov@oparina4.ru (O.I.); v_frankevich@oparina4.ru (V.F.); plotnikov@belozersky.msu.ru (E.P.); g_sukhikh@oparina4.ru (G.S.); 2 A.N. Belozersky Institute of Physico-Chemical Biology, Lomonosov Moscow State University, 119992 Moscow, Russia; 3 Moscow Institute of Physics and Technology, 141700 Moscow, Russia

**Keywords:** metabolomics, amino acids, diagnostics, neonatal asphyxia, liquid chromatography–mass spectrometry, newborn screening

## Abstract

Hypoxic–ischemic encephalopathy (HIE) is one of the most common causes of childhood disability. Hypothermic therapy is currently the only approved neuroprotective approach. However, early diagnosis of HIE can be challenging, especially in the first hours after birth when the decision to use hypothermic therapy is critical. Distinguishing HIE from other neonatal conditions, such as sepsis, becomes a significant problem in diagnosis. This study explored the utility of a metabolomic-based approach employing the NeoBase 2 MSMS kit to diagnose HIE using dry blood stains in a Rice–Vannucci model of HIE in rats. We evaluated the diagnostic fidelity of this approach in a range between 3 and 6 h after the onset of HIE, including in the context of systemic inflammation and concomitant hypothermic therapy. Discriminant analysis revealed several metabolite patterns associated with HIE. A logistic regression model using glycine levels achieved high diagnostic fidelity with areas under the receiver operating characteristic curve of 0.94 at 3 h and 0.96 at 6 h after the onset of HIE. In addition, orthogonal partial least squares discriminant analysis, which included five metabolites, achieved 100% sensitivity and 80% specificity within 3 h of HIE. These results highlight the significant potential of the NeoBase 2 MSMS kit for the early diagnosis of HIE and could improve patient management and outcomes in this serious illness.

## 1. Introduction

Hypoxic–ischemic encephalopathy (HIE) is a brain pathology which is typically manifested within the first hours of life. It is characterized by a complex spectrum of neurological disorders of different severity due to insufficient oxygen supply and low blood flow to the brain during childbirth. The incidence of severe HIE in the neonatal population is estimated at 0.37 to 3 cases per 1000 live births [1].

Several factors influence the prognosis of the severity of HIE, including the degree, location, and extent of structural brain damage caused by HIE. In general, the mortality rate associated with HIE is estimated to be between 9.1% and 9.9%, while in cases where the disease progresses to stage 3, the mortality rate can increase significantly to 70–80% [2]. It is important to acknowledge that about 25% of children with HIE suffer severe long-term neurological impairments, including cerebral palsy, seizures, mental retardation, cognitive impairment, and epilepsy. In addition, intellectual impairment and behavioral problems characterized by an intelligence quotient (IQ) of 70 or less are observed in a significant proportion of HIE cases, ranging from 50 to 80% [3,4,5,6]. These impairments can have an essential impact on the affected child’s cognitive development and overall quality of life. Therefore, accurate diagnosis, timely intervention, and appropriate treatment of HIE are crucial to minimize the risk of potential long-term adverse consequences of this pathology.

In the pathogenesis of HIE, phases of primary and secondary damage to nervous tissue are distinguished. Primary damage occurs during the period of asphyxia, and it is characterized by irreversible death of brain cells, the extent of which depends on the severity and duration of hypoxia. Secondary damage is activated in the reoxygenation-reperfusion phase 2–12 h after the primary damage. Secondary damage is caused by the activation of several pathogenetic mechanisms: glutamate and calcium stress, excessive free radical production, aseptic inflammatory processes, and activation of apoptosis, which lead to an increase in the volume of neural damage thus worsening the prognosis for life and health [7].

One of the promising approaches to reducing the adverse effects of central nervous system (CNS) injury is therapeutic hypothermia (TH), which is considered the most effective and safest neuroprotective method for infants who have suffered severe birth asphyxia [8,9]. It involves controlled chilling of the body to reach the core temperature as low as 33–34 °C for a specifically chosen time. Numerous experimental studies have shown that TH helps to reduce metabolic expenditures of the organism, secondary energy deficits in the cells, and the release of glutamate, also inhibiting free radical production and preventing inflammation and apoptosis [10,11]. 

Early and accurate assessment of the severity of HIE remains one of the greatest challenges in neonatal care [12,13]. However, modern methods of assessing the risk of brain injury in neonates have limitations associated with the first hours of life, and uncertainty about the severity of progressive brain injury and potential neurological consequences remains at this early stage [14]. For the early differential diagnosis of HIE in newborns, approaches to analyze metabolic changes in the blood of newborns using new analytical methods, in particular gas chromatography–mass spectrometry, may be suitable [15]. This method allows the identification of hundreds of metabolites within a few hours. Clinical studies during the neonatal period are very scarce. Non-invasive samples (such as urine and stool) and minimally invasive samples (such as blood spots) are preferential to study the dynamics of the molecular composition of biological fluids. However, infants with congenital neonatal asphyxia typically do not urinate for a long time due to kidney damage. Therefore, the main focus of research is on biomarkers of circulating blood [16]. A dried blood spot is the most promising and minimally invasive sample for the diagnosis of neonatal pathologies [17]. The analysis of amino acid and acylcarnitine levels in dried blood spots is a generally admitted approach for the screening of metabolic defects in newborns [18]. 

Recent advances in metabolomics, proteomics, and transcriptomics have focused the attention of researchers on the identification of comprehensive molecular signatures. These signatures may allow to precisely distinguish the effects of HIE in the first six hours of an infant’s life from other injuries commonly caused by perinatal asphyxia, such as renal and myocardial damage. Neonatal HIE is difficult to distinguish from sepsis or inborn errors of metabolism [16]. Sepsis may accompany HIE and alter the clinical features. It is important to point out that incorrect diagnosis and, accordingly, the appointment of hypothermia can be harmful to patients with non-ischemic encephalopathies [19]. This fact points to the need to seek markers of brain damage in neonates that are specifically associated with the ischemic/hypoxic genesis of the damage [20].

To simplify the multifactorial system in patients with HIE and to identify specific patterns of brain injury, proteomic and metabolomic analyses are used in in vivo and in vitro models. Using an ischemia-hypoxia model (carotid artery ligation and exposure to a mixed gas of 92% N_2_/8% O_2_ for 2 h), our recent study demonstrated the central role of glycerophospholipids, steroid biosynthesis, and fatty acid metabolism in triggering systemic responses in 7-day-old rats [21]. Other authors performed metabolomic studies of hypoxic–ischemic injury in neonatal pigs after hypoxia and 120 min of reoxygenation phase. During hypoxia, in blood plasma, the levels of metabolites indicating a transition to anaerobic metabolism, in particular cytidine and uridine derivatives, free fatty acids, and choline, increased significantly [22].

The persistent high morbidity and mortality in neonates with HIE underscores the urgent need for the introduction and implementation of improved diagnostic modalities. These advanced diagnostics are important not only to reliably confirm hypoxia/ischemia-induced complications but also to enable a rapid and prudent decision to initiate therapeutic hypothermia. In addition, such approaches have the potential to distinguish HIE and other clinical pathologies with phenotypically congruent symptoms, including but not limited to various inflammatory diseases. The integration of nuanced diagnostic measures is expected to improve the prognostic accuracy of HIE while streamlining the distinctive diagnostic processes to effectively distinguish HIE from a spectrum of alternative illnesses.

A routine neonatal screening platform using the non-derivatized MSMS kit NeoBase 2 (Perkin Elmer, Turku, Finland), which can detect 57 metabolites and more than 30 different metabolic diseases by tandem mass spectrometry (MSMS), was used.

## 2. Results

**Figure 1:**
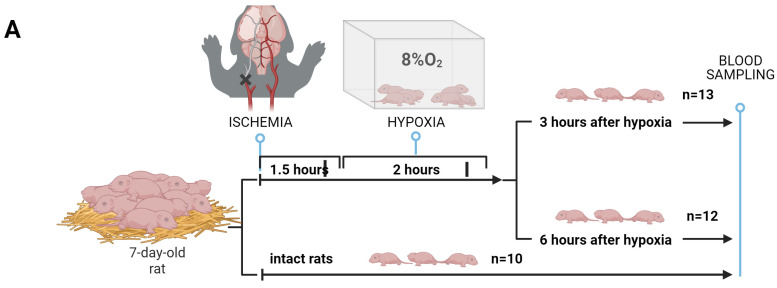

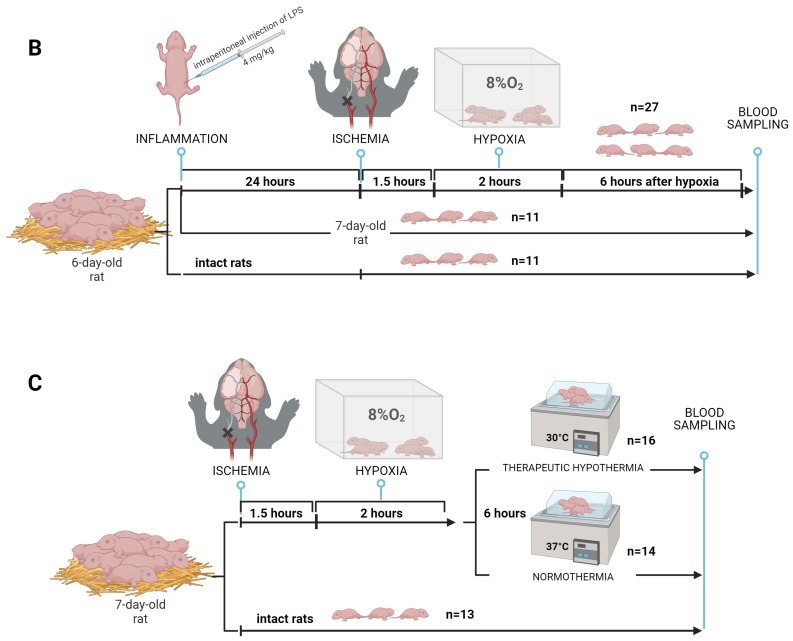
Study design: (**A**) HIE with sampling at different times; (**B**) HIE model supplemented with LPS-induced inflammation; (**C**) modeling of therapeutic hypothermia.

To search for HIE-specific markers of brain damage and to determine metabolic changes in the blood during therapeutic hypothermia or systemic inflammation, three different model experiments were performed (Figure 1). The first goal was to evaluate the changes in the blood of 57 compounds of low molecular mass (14 amino acids, 36 carnitines, 2 nucleosides, succinylacetone, and 4 lysophosphatidylcholines) over time: before HIE and 3 and 6 h after HIE. In the second part, the specificity of potential markers of HIE related to acute inflammation (lipopolysaccharide (LPS)-induced) was explored. A third set of experiments was performed to reveal the effects of hypothermia on the molecular profile of the blood. Quantitative FIA-MRM-MS analysis (flow injection multiply reaction monitoring mass spectrometry analysis) of 57 metabolites in a dry blood spot (DBS) was performed as part of routine neonatal screening, which could make the results obtained in this study as close as possible to clinical practice.

### 2.1. Time-Related Changes in DBS after Hypoxia–Ischemia

**Figure 2:**
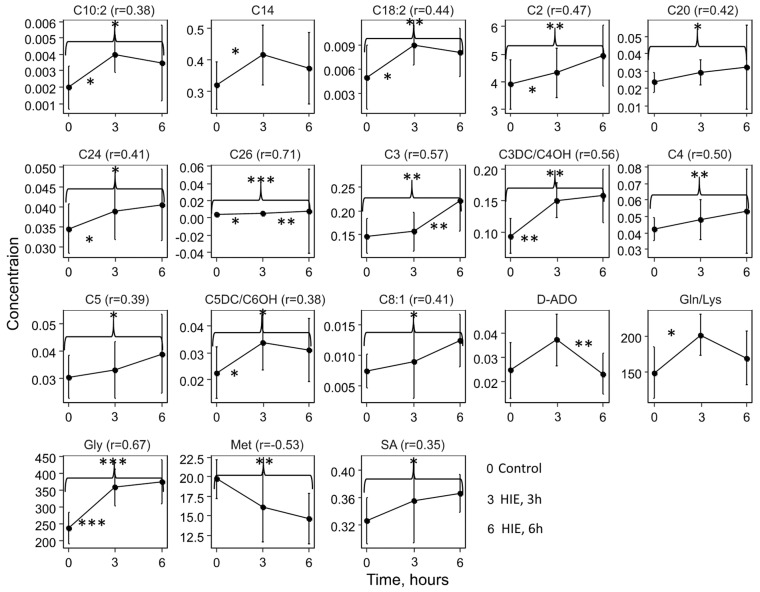
Line graphs representing the concentration levels of metabolites that show significant changes over time. Data are presented as mean  ±  SD. *—*p* < 0.05, **—*p* < 0.01, ***—*p* < 0.001—significance changes between neighbor timepoints or, in case of curly brackets between control (0) and HIE, 6 h (6) according to the Mann–Whitney test.

Three hours after hypoxia, a statistically significant increase in the levels of glycine, glutamine/lysine, and acylcarnitines: C3DC/C4OH (malonylcarnitine/3-hydroxybutyrylcarnitine), C5DC/C6OH (glutarylcarnitine/3-hydroxyhexanoylcarnitine), C10:2 (decadienoylcarnitine), C26 (hexacosanoylcarnitine), C18:2 (octadecadienoylcarnitine), C14 (tetradecanoylcarnitine), and C24 (lignoceroylcarnitine) is observed. However, only the differences in glycine levels remained statistically significant after FDR (false discovery rate) correction (FDR = 0.004) (Table S1). Six hours after the induced damage, changes accumulate and are primarily associated with an increase in glycine levels, succinylacetone, acylcarnitines: C26, C3DC/C4OH, C3 (propionylcarnitine), C4 (butyrylcarnitine), C18:2, C2 (acetylcarnitine), C5DC/C6OH, C24, C8:1 (2-octenoylcarnitine), C20 (arachidoylcarnitine), C5 (valerylcarnitine), and C10:2, and a decrease in methionine levels (Table S2). The levels of acylcarnitines C26 and C3 were significantly increased, while the level of deoxyadenosine decreased after 3 and 6 h of hypoxia–ischemia. Figure 2 illustrates the metabolite levels in DBS, with statistically significant changes in at least one of the compared groups: control (0 h), 3 h, and 6 h after hypoxia–ischemia.

**Figure 3:**
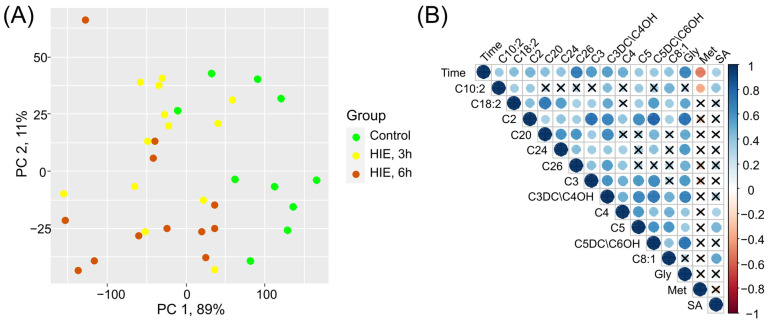
The impact of time after hypoxic–ischemic exposure on the low-molecular-weight spectrum of DBS: (**A**) Principal Component Analysis (PCA) space based on blood compounds, whose levels show statistically significant differences in pairwise group comparisons; (**B**) Correlation diagram of the compounds that show statistically significant associations with the time elapsed since the HIE. X is a label indicating the absence of a significant association.

In the principal component space, samples from the control group and samples were taken 6 h after hypoxia are distinctly separated, while 3 h samples occupy an intermediate position (Figure 3A). The levels of glycine, succinylacetone, and acylcarnitines C10:2, C18:2, C2, C20, C24, C26, C3, C3DC/C4OH, C4, C5, C5DC/C6OH, and C8:1 significantly increase over time after hypoxia, while the level of methionine decreases (Figure 3B).

According to the SMPDB (small molecular pathway database) library, three hours after ischemia/hypoxia, there was a statistically significant (*p* < 0.05) enrichment in several pathways, including the ammonia cycle, beta-oxidation of long-chain fatty acids, alanine metabolism, and carnitine metabolism. Additionally, according to the KEGG (Kyoto Encyclopedia of Genes and Genomes) library, pathways such as glyoxylate and dicarboxylate metabolism, aminoacyl-tRNA (aminoacyl-transfer-ribonucleic acid) biosynthesis, glutathione metabolism, and porphyrin and chlorophyll metabolism were enriched (Table S3).

According to the SMPDB library, based on enriched statistically significant markers identified six hours after hypoxia/ischemia, possible activation of pathways such as branched-chain fatty acid oxidation, methionine metabolism, glycine, and serine metabolism was suggested. According to the KEGG library, pathways such as aminoacyl-tRNA biosynthesis, glutathione metabolism, porphyrin and chlorophyll metabolism, glyoxylate and dicarboxylate metabolism, glycine, serine, and threonine metabolism, and cysteine and methionine metabolism were also identified as changed (Table S4).

### 2.2. The Influence of Acute Inflammation (LPS-Induced) and Hypoxia–Ischemia on DBS Metabolome 

**Figure 4:**
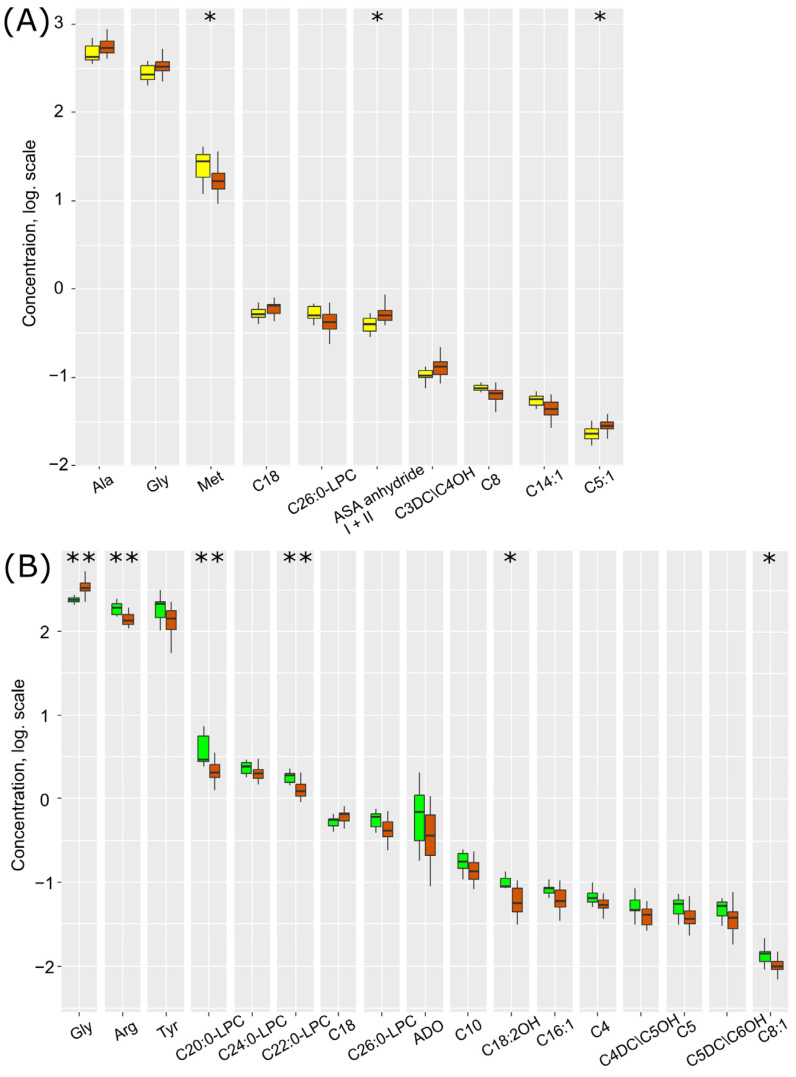
Box plots of DBS metabolites, statistically significantly differing (*p* < 0.05 according to the Mann–Whitney test) in comparisons between (**A**) LPS-induced inflammation (yellow) and inflammation associated with HIE (sienna) and (**B**) control (green) and inflammation associated with HIE (sienna). *—*p* < 0.01, **—*p* < 0.001 according to the Mann–Whitney test.

Figure 4A illustrates the levels of compounds demonstrating a statistically significant difference. With the exacerbation of the inflammatory process by ischemia/hypoxia, there is a profound increase in the levels of argininosuccinic acid anhydrides, glycine, alanine, and acylcarnitines C5:1 (tiglylcarnitine), C3DC/C4OH, and C18 (octadecanoylcarnitine), and a decrease in the levels of methionine, lysophosphatidylcholine C26:0, and acylcarnitines C8 (octanoylcarnitine) and C14:1 (tetradecenoylcarnitine).

In the principal component space, the control group occupies a separate cluster (Figure S1). Two clusters of compounds can be identified with apparent differences between the inflammation group and the inflammation with ischemia/hypoxia group (Figure S2).

Thus, out of the 18 compounds identified as potential markers of hypoxic–ischemic injury (Figure 2), 12 (67%) are specific in the context of the inflammatory process. Short-chain acylcarnitines C4, C5, C5DC/C6OH, and C8:1 show a statistically significant decrease in hypoxic–ischemic damage (Figure 4B). This bidirectional change suggests the utility of these metabolites in determining the inflammatory background. Glycine is significantly elevated both in hypoxia–ischemia and in inflammation with hypoxia–ischemia. Conversely, methionine decreases in hypoxia–ischemia independently of inflammation as a comorbidity.

According to the SMPDB library, in inflammation coupled with hypoxia–ischemia, relative to inflammation alone, glycine and serine metabolism, alanine metabolism, glutathione metabolism, methionine metabolism, glutamate metabolism, glucose–alanine cycle, spermidine and spermine biosynthesis, and betaine metabolism were statistically significantly changed. For glycine and serine metabolism, alanine metabolism, glutathione metabolism, and methionine metabolism, as well as glutamate metabolism, the average significance of random enrichment was *p* < 0.05 (Figure S3A). According to the KEGG library, statistically significant over-representation was observed in pathways such as aminoacyl-tRNA biosynthesis; selenium compound metabolism; alanine, aspartate, and glutamate metabolism; glutathione metabolism; porphyrin and chlorophyll metabolism; glyoxylate and dicarboxylate metabolism; glycine, serine, and threonine metabolism; cysteine and methionine metabolism; and the biosynthesis of primary bile acids. In this case, aminoacyl-tRNA biosynthesis and selenium compound metabolism have a significance of random enrichment of less than 5% (*p* < 0.05) (Figure S3B).

### 2.3. Therapeutic Effect of Hypothermia in Hypoxic–Ischemic Injury

**Figure 5:**
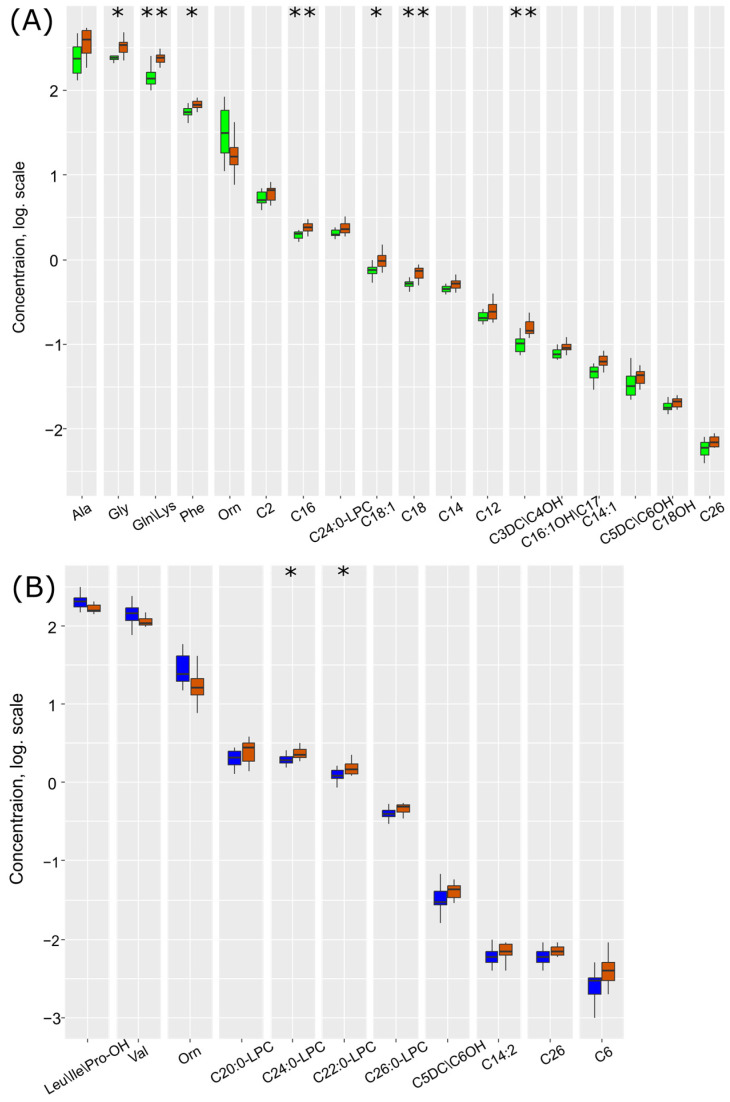
Statistically significantly changed metabolites (*p* < 0.05 according to the Mann–Whitney test) between (**A**) control group (green) and HIE with 6 h of normothermic mode (sienna), and (**B**) HIE with 6 h of TH mode (blue) and HIE with 6 h of normothermic mode (sienna). *—*p* < 0.01, **—*p* < 0.001 according to the Mann–Whitney test.

In the third set of experiments, one-third of the rats after hypoxia–ischemia were exposed to 6 h of hypothermia. During this, the DBS metabolome also demonstrated significant changes compared to normothermia and the control group (Figure 5A). Under the hypothermic mode after hypoxia–ischemia, there was a statistically significant increase in the levels of ornithine, valine, and leucine/isoleucine/hydroxyproline and a statistically significant decrease in the levels of lysophosphatidylcholines C20:0, C22:0, C24:0, C26:0, and acylcarnitines C26, C5DC/C6OH, C6 (hexanoylcarnitine), and C14:2 (tetradecadienoylcarnitine) when compared with the normothermic mode after hypoxia–ischemia (Figure 5B, Table S5). Similar to hypoxic–ischemic injury amid the inflammatory process, the DBS metabolites are divided into two clusters, with connections differing between the normothermia group and the hypothermia group (Figure S4).

According to the KEGG library, under the hypothermic mode, compared to normothermia, there was a statistically significant enrichment in the pathways of valine, leucine, and isoleucine biosynthesis and degradation; aminoacyl-tRNA biosynthesis; arginine biosynthesis; pantothenate and CoA (coenzyme A) biosynthesis; arginine and proline metabolism; and glutathione metabolism. All pathways, except the last one, have a significance of random enrichment of *p* < 0.05.

### 2.4. Development of the Diagnostic Model for HIE

**Figure 6:**
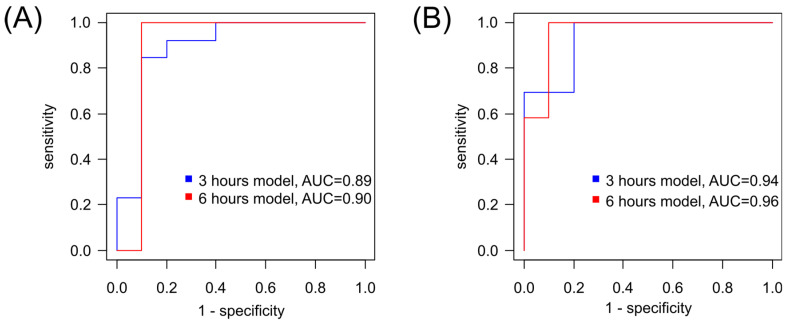
Receiver operating characteristic (ROC) curves for diagnosing after 3 h and 6 h post-HIE based on (**A**) logistic regression models, which utilize the concentration of glycine raised to the second power [Gly]^2^ as a variable, or (**B**) orthogonal partial least squares (OPLS) models.

The logistic regression model for diagnosing hypoxia–ischemia after 3 h of exposure has a sensitivity of 85% and specificity of 90%, with a cutoff threshold of 0.57 (Figure 6A, Table S6). The model for diagnosing ischemia/hypoxia after 6 h of exposure has a sensitivity of 100% and specificity of 90% with a cutoff threshold of 0.39 (Figure 6A, Table S7). For both models, glycine emerged as the only marker compound.

**Figure 7:**
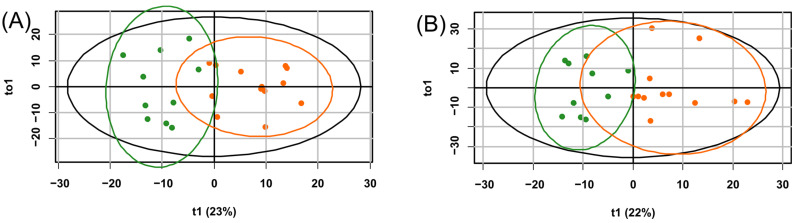
OPLS-DA scores plot for the diagnosis of hypoxia after (**A**) 3 h and (**B**) 6 h of exposure. The control group is indicated in green, and the group with hypoxic–ischemic injury is shown in orange.

Additionally, for the diagnosis of hypoxia–ischemia based on the plasma profile 3 h after the presumed event, an orthogonal partial least squares discriminant analysis (OPLS-DA) model with two orthogonal projections was constructed (Figure 7A). The proportion of described independent variables, X, was 56%, described dependent variables, Y, constituted 74%, and the proportion of predicted dependent variables, Y, was 63%. Based on cross-validation results, the model demonstrated a sensitivity of 100% and specificity of 80% for the 3 h timeframe, with a cutoff threshold of 0.38 (Figure 6B). Key markers for hypoxia (VIP (variables importance projection) > 1) included glycine, alanine, glutamine/lysine, tyrosine, and ornithine (Figures S5 and S6).

A 6-h hypoxia–ischemia OPLS-DA diagnostic model with four orthogonal projections was also constructed (Figure 7B) with the following performance parameters: the proportion of described independent variables, X, was 68%; described dependent variables, Y, constituted 68%; and the proportion of predicted dependent variables, Y, was 57%. According to cross-validation results, the model demonstrated a sensitivity and specificity of 100% and 90%, respectively, with a cutoff threshold of 0.49 (Figure 6B). Key markers for hypoxia (VIP > 1) included glycine, glutamate, leucine/isoleucine/hydroxyproline, citrulline, phenylalanine, glutamine/lysine, and valine (Figures S7 and S8).

Both models exhibit good diagnostic potential, with the last diagnostic model having higher specificity. For both models, the key compounds are glycine and glutamine/lysine.

To assess the diagnostic accuracy of the developed models (OPLS and logistic regression) using metabolite levels in DBS after 6 h of hypoxia–ischemia, the data obtained in the third experiment were utilized: a control group (n = 13) and a normothermia hypoxia–ischemia group (n = 14). Validation based on 100-times split cross-validation of samples from the first and the third sets of experiments demonstrated a sensitivity of 86% and specificity of 88% for the OPLS model and a sensitivity of 87% and specificity of 74% for the logistic regression model.

## 3. Discussion

The diagnosis of HIE is a major challenge due to limitations in current clinical methodology and tools [14,23,24]. Although amplitude-integrated electroencephalography (EEG) and imaging devices such as magnetic resonance imaging (MRI) are common techniques for predicting long-term outcomes and assessing the severity of brain injury, they often fall short in the critical first hours of life when rapid and accurate diagnosis is highly needed to make a decision whether to use TH [12,13,25]. In addition, there are factors such as the need for continuous EEG monitoring to detect subclinical seizures that may be clinically unnoticed and the possibility that neuroimaging may underestimate brain injury if performed too soon after the hypoxic challenge [26]. In addition, comorbidities, including perinatal sepsis and metabolic disorders, may interfere with the neuroprotective effect of TH and complicate the diagnostic process [27,28,29]. Although metabolic defects in inborn are rare, they may demonstrate symptoms like those under HIE [30,31].

In this complex diagnostic landscape, the analysis of metabolic markers might be a promising approach [32]. Metabolomics offers a nuanced view of the biochemical changes that occur as a result of HIE and can provide earlier and more precise indications of damage compared to conventional imaging and neurophysiological studies [33,34]. This methodology is particularly valuable in distinguishing between HIE and other disorders with similar manifestations, such as inborn metabolic defects or genetic encephalopathies, which can mimic HIE symptoms [35]. Consideration of detailed family history and targeted metabolic testing are critical to identifying the conditions that require treatment other than those used in HIE, including specific treatments instead of TH. With advances in neonatal care research, the integration of metabolomic data into existing diagnostic tools holds the potential to increase the accuracy of HIE diagnoses and personalize interventions to improve outcomes for affected infants.

Although metabolomics is a promising approach for the diagnosis of HIE, it is important to note that many of the approaches being investigated in this area are still used in the experimental mode and require further standardization before they can be used in clinical practice. However, there are approaches that have already been used in clinical practice, such as the NeoBase 2 MSMS kit, which is currently employed in newborn screening [36]. It allows the simultaneous analysis of a comprehensive panel of a total of 57 metabolites involved in different metabolic pathways, such as amino acids, carnitines, nucleosides, and lysophosphatidylcholines. The main limitation of this set in the context of searching for HIE markers is, of course, the small number of metabolites compared to standard mass spectrometry. However, we assumed that this set of metabolites would be sufficient for the differential diagnosis of HIE. In particular, the applicability of this kit for the diagnosis of various pathological conditions in addition to conventional newborn screening has already been demonstrated [37,38,39]. Also, the NeoBase 2 MSMS kit takes advantage of the convenience and practicality of analyzing dried blood spots on paper, which are widely available and commonly used as a source of neonatal biological samples [40]. This eliminates the need for invasive procedures and simplifies sample collection, transportation, and storage.

Research on HIE markers is constrained by strict ethical standards associated with neonatal studies and the low incidence of HIE, limiting the clinical research capacity to fully validate diagnostic tests. To overcome these hurdles, experimental animal models of HIE are invaluable as they provide the opportunity to study the pathogenetic mechanisms and markers of brain injury in a precisely controlled environment. In our study, we used the Rice–Vannucci model, a widely recognized experimental model that closely mimics the pathophysiological changes observed in neonates with HIE [41]. We have also used one of the most common models of neonatal sepsis [42], which complicates the diagnosis of HIE and may also be a contraindication to hypothermic therapy when both sepsis and HIE are present. Additionally, we assessed how TH impacts the profile of markers, particularly focusing on prognostic implications.

In all three experiments, we observed a statistically significant increase in the levels of the acylcarnitines, mainly short-chain acylcarnitine. Acylcarnitines are formed by the esterification of carnitine, which is needed to transport fatty acids through the mitochondrial membrane for subsequent beta-oxidation, yielding energy. Under hypoxia–ischemia, energy production in the cell is disturbed, which leads to a change in metabolism and redirection of bioenergetics toward anaerobic glycolysis. This, in turn, affects the levels of various metabolites, including acylcarnitines. The accumulation of certain acylcarnitines may reflect a disturbance in mitochondrial function and energy metabolism. Clinical studies demonstrated that changes in the acylcarnitine profile can serve as a biomarker for HIE. A study examining a cohort of 67 children diagnosed with HIE revealed that butyrylcarnitine (C4) could serve as a potential prognostic biomarker. The observed correlations between circulating C4 levels, neuron-specific enolase concentrations, and MRI abnormalities suggest that this biomarker is useful for the prognostic assessment of HIE [43]. Analysis of umbilical cord blood showed elevated concentrations of long-chain acylcarnitines in two different groups of newborns: those who had clinical symptoms of hypoxia–ischemia damage but had a normal neurological examination and those who were diagnosed with HIE [44]. Another study in which newborns were diagnosed with HIE revealed elevated levels of acylcarnitines, specifically palmitoylcarnitine (C16:0), stearoylcarnitine (C18:0), and oleoylcarnitine (C18:1), which were correlated with more acidic pH values in the umbilical artery and lower Apgar scores five minutes after delivery [45]. Dave and co-authors explored the tissue-specific origin of acylcarnitines using a mouse model of HIE and quantified these metabolites in skeletal muscle, brain, and blood. They found that acylcarnitines C16:0 and C18:0 increase in plasma as well as in brain tissue 30 min after HIE and dissipate after 24 h. On the other hand, all other acylcarnitines were elevated only in muscle and plasma. The authors made conclusions that plasma acylcarnitine profiles predominantly reflect muscle-derived changes rather than those occurring in the brain [46]. In our study, after applying the FDR correction, acylcarnitines were excluded as reliable biomarkers for the diagnosis of HIE, although they were initially statistically significant. Due to the predominant skeletal muscle origin of acylcarnitines following systemic hypoxic/ischemic events, the early post-HIE elevation in their levels may be more indicative of the presence of systemic hypoxia rather than the index of the extent of the cerebral injury. Consequently, acylcarnitines could serve as markers of systemic hypoxia in neonates and indirectly point to possible injury to the brain, the organ most susceptible to oxygen deficiency. However, comprehensive, long-term, multi-center studies of changes in acylcarnitine levels in neonates with HIE are needed for definitive conclusions.

In modeling a systemic inflammatory process associated with HIE, our study found a change in the levels of metabolites responsible for several dysfunctions, including decreased oxygenation, excitotoxicity, impaired glucose utilization, impaired fatty acid metabolism, impaired protein synthesis, deterioration of cell membrane integrity, mitochondrial dysfunction, and impaired energy production. We found a decrease in short-chain acylcarnitine levels during hypoxic–ischemic injury followed by an increase associated with an increase in the inflammatory response, which could indicate carnitine deficiency. Carnitine deficiency is a contributing factor to the progression of inflammatory processes [47]. Although the analysis involving a panel of 57 metabolites provided insights into the pathophysiological processes, principal component analysis failed to distinguish between groups subjected to 24 h LPS treatment and those modeled with HIE concurrent with systemic inflammation, as significant clustering was only observed within the control group. More studies are needed employing wider sets of metabolites and various analytical methods for distinguishing these two conditions.

In the context of hypothermia after HIE, we observed a significant decrease in markers associated with HIE, such as acylcarnitines and the amino acids identified in the OPLS-DA diagnostic model. In addition, we observed significant changes in metabolic pathways responsible for ammonia detoxification, reduced lipid peroxidation activity, and stabilization of cellular energy metabolism after TH. These results suggest a positive effect of TH on biochemical processes and recovery mechanisms after HIE, which is consistent with previous studies in this field [48,49]. Furthermore, our results emphasize the sensitivity of the investigated markers to the extent of brain damage after HIE. Considering these results, we recommend using the NeoBase 2 MSMS kit for exploring neuroprotective strategies in animal models during the acute phase of the disease.

In this study, OPLS-DA and logistic regression models with high diagnostic accuracy were developed for the timely diagnosis of HIE. Moreover, 3- and 6-h models were similar with respect to included variables and performance. The models based on the DBS profile at six hours were validated using a new set of samples from experiment 3. Validation demonstrated sensitivity and specificity of more than 86%. Interestingly, glycine was the only DBS compound included in logistic 3- and 6-h regression models. Its VIP was the highest in both OPLS-DA models, which also included various amino acids. These compounds may be suggested as HIE markers in DBS. The main marker for HIE that we have identified is glycine, which is consistent with clinical data for newborns with HIE [50,51]. Also, an increase in glycine has been described for models of global brain ischemia [51,52,53] and for patients in the acute phase of stroke in both plasma and cerebrospinal fluid [54,55]. We suggest that the main source of glycine could be a product of reduced nicotinamide adenine dinucleotide (NADH) synthesis reaction from serine under hypoxic conditions [56]. NADH plays a vital role in ATP synthesis. Independently of the tricarboxylic acid cycle, mitochondrial serine catabolism generates NADH through a folate-dependent mechanism. The enzyme serine hydroxymethyltransferase 2 catalyzes the conversion of serine to glycine, concurrently generating 5,10-methylene tetrahydrofolate (methylene-THF). Subsequently, methylene tetrahydrofolate dehydrogenase 2 oxidizes methylene-THF, with a reduction of NAD+ and the formation of 10-formyl-THF. It is also important to consider that hypoxic/ischemic conditions can disrupt neurotransmitter homeostasis, potentially leading to extracellular accumulation of neurotransmitters, including glycine, which can subsequently be released into the bloodstream [54]. This phenomenon has been observed particularly in the ischemic stroke model, with the increase in glycine concentration correlating with the extent of injury [57].

In addition to glycine, the OPLS-DA diagnostic model also includes glutamate, leucine, isoleucine, hydroxyproline, citrulline, phenylalanine, and the combined markers glutamine/lysine and valine as main markers of HIE. In particular, glutamate is known to be a key excitatory neurotransmitter in the brain, which plays an important role in acute neurological conditions, including HIE [58]. In neonatal HIE, there is a critical disturbance of the brain’s energy metabolism. The interruption of oxygen and glucose supply may impede oxidative production of ATP and lead to a failure of ATP-dependent ions pumping. This may lead to a pathological reversal of the functions of the glutamate transporter functions, resulting in an excessive release of glutamate into the extracellular space. The result is a chain reaction of excitotoxicity that exacerbates neuronal damage and is reflected in increased plasma glutamate concentrations [59]. In addition, hypoxia/ischemia leads to a metabolic shift toward anaerobic metabolic pathways, disabling the Krebs cycle and leading to an accumulation of cycle intermediates and byproducts of pyruvate metabolism, potentially increasing the concentrations of precursor amino acids identified in our study. The role of amino acids as biomarkers for early HIE has previously been demonstrated in both model experiments and clinical studies [15,44,60,61,62]. Furthermore, these amino acids could serve as alternative energy substrates for the neonatal brain and muscles under hypoxic conditions [63]. It is important to note that the enrichment of metabolic pathways that we identified 3 and 6 h after HIE indicates the response of the brain and entire organism to hypoxic/ischemic damage, including a transition to anaerobic metabolism, compromised cellular energy production, perturbed glucose metabolism, and protein biosynthesis and disrupted reparation processes. The diagnostic biomarkers we have identified are intrinsically linked to these early pathophysiological events and are pathogenetically relevant. They denote the initiation of a patho-biochemical cascade that underlies the cerebral damage associated with HIE. Therefore, these markers may serve as important indicators of early brain injury and assist in guiding therapeutic interventions during the initial phase of HIE.

We should note that the NeoBase 2 kit was originally validated for clinical studies in humans, especially for newborn screening, but not for animal studies. However, the use of the NeoBase 2 kit in experimental research on animal models does not require the same validation as in the clinical setting. Since the aim of our study was not to determine the absolute concentrations of the analyzed substances but to compare metabolite profiles between the experimental and control groups, the accuracy of the kit is less important than in a clinical diagnosis based on absolute values. This approach is consistent with established methods in comparative biomedical research. We also admit that metabolic differences between rats and humans may limit the direct translation to humans of biomarkers detected in rats. To address this issue in the context of modeling HIE, we used the Rice–Vannucci model, which closely simulates the pathogenetic mechanisms observed in neonates with this condition [41]. Previous research has shown that many acute brain pathologies in humans and rodents produce similar metabolites due to shared pathobiochemical processes [64]. The relevance of metabolites identified in our study was confirmed by the data from other studies. Moreover, metabolites such as glycine, glutamate, and acylcarnitines were validated as associated with HIE in neonates in clinical studies.

## 4. Materials and Methods

### 4.1. Animals

The animal protocols performed were reviewed and approved by the institutional animal ethics committee according to the guidelines of FELASA (Federation of European Laboratory Animal Science Associations). The experiments were performed with outbred white rats obtained from the animal facility of the A.N. Belozersky Institute of Physico-Chemical Biology. To ensure animal welfare, dams and their pups were housed in cages in a temperature-controlled environment with a temperature of 21 ± 2 °C. In addition, a light–dark cycle was established in which the light was switched on from 9:00 a.m. to 9:00 p.m. The dams had ad libitum access to food and water, and pups were examined daily for health status.

### 4.2. HIE Modeling

The study used the well-established Rice–Vannucci rat model to investigate hypoxic–ischemic brain injury (HIE). The experiments were conducted with seven-day-old postnatal rats of both sexes. The left carotid artery was surgically isolated and obliterated with an electrocautery to deliberately restrict the blood supply to the brain. The rats were then exposed to a gas mixture of 8% oxygen and 92% nitrogen for a defined period of 2 h in a multigas CO_2_ incubator (Binder, Tuttlingen, Germany) at a temperature of 37 °C, starting 1.5 h after surgery, to induce hypoxia. The mortality rate after hypoxia–ischemia induction was in a range of 3–5%. The experiments were conducted to explore different impacts on rat pups with HIE, which were divided into experimental groups as follows.

#### 4.2.1. HIE with Sampling at Different Times

Pups were randomly divided into the following experimental groups: intact rats (n = 10), animals with HIE whose blood samples were taken 3 h after hypoxia (n = 13), and animals with HIE whose blood samples were taken 6 h after hypoxia (n = 12) (Figure 1A). Three and six hours after the hypoxic exposure, dry blood spots were obtained.

#### 4.2.2. HIE Model with LPS-Induced Inflammation

In this set of experiments, 6-day-old rat pups were injected intraperitoneally with 4 mg/kg body weight of lipopolysaccharide (LPS) 24 h before the induction of hypoxia and ischemia. The animals were divided into three experimental groups: intact rats (n = 11), rats injected with LPS without HIE (n = 11), and rats injected with LPS with HIE (n = 27) (Figure 1B). After 6 h of hypoxic exposure, dry blood spots and blood plasma were collected for analysis.

#### 4.2.3. Modeling of Therapeutic Hypothermia

Pups were randomly assigned to either normothermic or hypothermic recovery immediately after termination of hypoxia for a duration of 6 h. Normothermic recovery was maintained at a temperature of 37 °C, while hypothermic recovery was maintained at 30 °C according to the previously described protocol [65]. Pups were placed in an open container in a water bath to achieve the desired temperatures. In this experimental set, there were three groups of animals: intact rats (n = 13), rats with HIE and hypothermia (n = 16), and rats with HIE and normothermia (n = 14) (Figure 1C). As in the previous experiments, dry blood spots (DBS) were collected after 6 h of hypoxic exposure for further analysis.

### 4.3. Dried Blood Spot Metabolome Analysis (FIA-MRM-MS)

The levels of 57 low-molecular-weight compounds, including 14 amino acids, 36 carnitines, 2 nucleosides, succinylacetone, and 4 lysophosphatidylcholines (Table S7), were measured in DBS using the flow injection analysis tandem mass spectrometry (FIA-MS/MS) as a part of a routine newborn screening at the Clinical Diagnostic Laboratory of the National Medical Research Center for Obstetrics Gynecology and Perinatology named after Academician V.I. Kulakov of the Ministry of Healthcare of the Russian Federation. Experiments were performed on UHPLC QSight MD autosampler coupled to a QSight 225MD tandem mass spectrometer (MSMS) in a positive electrospray (ESI) mode using multiple reaction monitoring (MRM) for signal acquisition. The NeoBase 2 non-derivatized MSMS kit #3044-0010 (Perkin Elmer, Turku, Finland) was applied to extract and quantitatively analyze target compounds in DBS. The kit includes NeoBase 2 Internal Standards, Extraction Working Solution (EWS), NeoBase 2 Controls (Low and High), Neo MSMS Flow Solvent, NeoBase 2 Extraction Solution, and NeoBase 2 Succinylacetone Assay Solution. NeoBase 2 assay was assessed based on the recommendations of the CLSI (the Clinical and Laboratory Standards Institute) guidelines [66]. Several studies confirmed good analytical performance of Neobase 2 kit in terms of precision, accuracy, limit of detection (LOD), lower limit of quantification (LLOQ), linearity, recovery, and carryover [67,68,69,70].

All analytical steps were performed in accordance with the manufacturer’s guidelines. In brief, a 3.2 mm disc was automatically punched from the DBS of each sample into a 96-well plate using Panthera-Pancher 9 (Perkin Elmer, Turku, Finland). DBS spots were incubated in the shaker with 125 µL of the extraction working solution (EWS), including internal standards (IS), for 30 min at +45 °C. Then, 100 µL supernatant was transferred to another plate’s well for 60 min incubation at room temperature. The 10 µL injection into the ion source was performed under the following conditions: capillary voltage of 5.5 kV, source temperature of 120 °C, hot source induced desolvation (HSID) temperature of 320 °C, drying gas flow of 80 L/h, and nebulizer gas flow of 120 L/h. FIA-MRM-MS was performed at solvent flow rate of 1 µL/s with duration run of 1.5 min and scanning rate of 1.76 s/scan. MRM parameters are listed in Table S8. The low and high level controls (LC, HC) were run at the start and the end of in each plate. The mean measured QCs were within ± 3 SD of the values provided by the quality control certificate. Processing of the data and analytes concentration calculation was carried out by Simplicity 3Q MD 2.1 software (Perkin Elmer, Turku, Finland). Result of mass-spectrometry analysis containing in Table S9.

Figure S9 presents an example of a typical flow injection chromatogram obtained for an LC sample provided by NeoBase™ 2 Non-derivatized MSMS kit on the QSight 225MD tandem mass spectrometer (PerkinElmer, Waltham, MA, USA).

### 4.4. Statistical Methods

The statistical analysis was performed by R 4.3.2 language scripts in RStudio space 23.09.1 version. 

The groups were compared pairwise by the Mann–Whitney test, and *p*-values were corrected using the Benjamin–Hochberg method (false discovery rate, FDR). A *p*-value less than 0.05 was considered statistically significant. Compounds with statistically significant differences in levels were used for principal component analysis (PCA).

To investigate the correlation between the time elapsed since hypoxic–ischemic brain injury and the molecular composition of blood, a Spearman test was applied with criteria of significant *p* < 0.05.

For each task, a pairwise comparative analysis of interactions between compounds within groups was performed: a correlation matrix and an adjacency change matrix were calculated for two groups. Based on the adjacency change matrix, a difference matrix was computed for hierarchical cluster analysis using Ward’s method. The optimal number and composition of clusters were determined based on the maximum silhouette coefficient [71].

Pathway enrichment analysis was performed using the over-representation analysis in the MetabolanalystR 4.0.0 package. A result with *p* < 0.1 was considered statistically significant. If a marker set contained compounds with multiple identification variants, new variable sets were created for each identification variant, each containing one of the options. In this case, pathways were considered statistically significant if *p* was less than 0.1 for each marker set. Enrichment values and probability of observing were averaged across all marker sets, and the median value was calculated for the number of markers in each pathway.

For hypoxia diagnosis, classification models were built based on OPLS-DA. Sensitivity and specificity were determined based on leave-one-out cross-validation results.

For diagnostic models based on logistic regression, a new dataset was created, including metabolite concentrations, the product of concentrations for each pair of metabolites, and the squares of the concentration values for each metabolite. In the preliminary marker set, variables with a variable projection value greater than 1 in orthogonal projections analysis were selected. A stepwise variable selection was performed based on the Akaike information criterion to obtain the minimum criterion value at each stage, while observing a decrease in the criterion value. Subsequently, a stepwise exclusion of variables was carried out, with coefficients having a maximum probability of zero equality until coefficients for variables had a probability of zero less than 0.05. The model’s quality was evaluated based on leave-one-out cross-validation.

Additional validation of 6 h’ models (OPLS and logistic regression) was performed by 100-times cross-validation using train/test split (70/30) of first experiment samples (Figure 1A, control (n = 10) and 6 h after hypoxia–ischemia (n = 12) groups) with addition of samples from the third experiment (Figure 1C, control (n = 13) and normothermic hypoxia–ischemia (n = 14) groups).

## 5. Conclusions

The diagnostic models that we developed using the NeoBase 2 MSMS kit demonstrate the possibility of diagnosing HIE as early as 3 h after disease onset. The relatively short duration (approximately 3 h) of the analysis fits well with the critical time for initiating TH that can mitigate the long-term effects of HIE. A study based on neonates will be promising for exploring the potential of early HIE diagnosis based on the DBS metabolome.

## Data Availability

Data are contained within the article and Supplementary Materials.

## Supplementary Materials

The supporting information can be downloaded at: https://www.mdpi.com/article/10.3390/ijms25042035/s1.
