# Supplementary material for: Metabolite Biomarkers for Early Ischemic–Hypoxic Encephalopathy: An Experimental Study Using the NeoBase 2 MSMS Kit in a Rat Model
Source: Int J Mol Sci. 2024 Feb 7;25(4):2035. doi: 10.3390/ijms25042035 (PMC10888647; doi:10.3390/ijms25042035)
Supplement: Supplementary file 1 [file ijms-25-02035-s001.zip › ijms-2782235-supplementary/Supplementary Figures and tables.pdf]

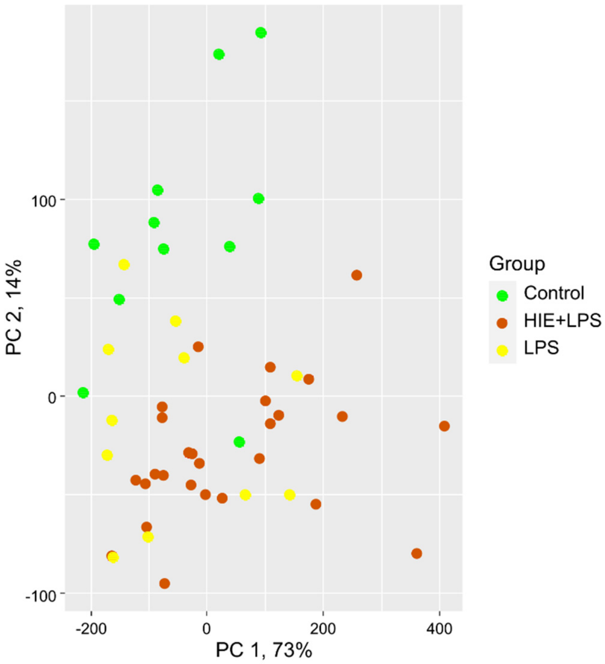


**Figure S1.** Principal component analysis (PCA) for the study of the effect inflammation influence on ischemia/hypoxia DBS metabolites, whose levels are statistically significantly different when comparing pairwise groups.

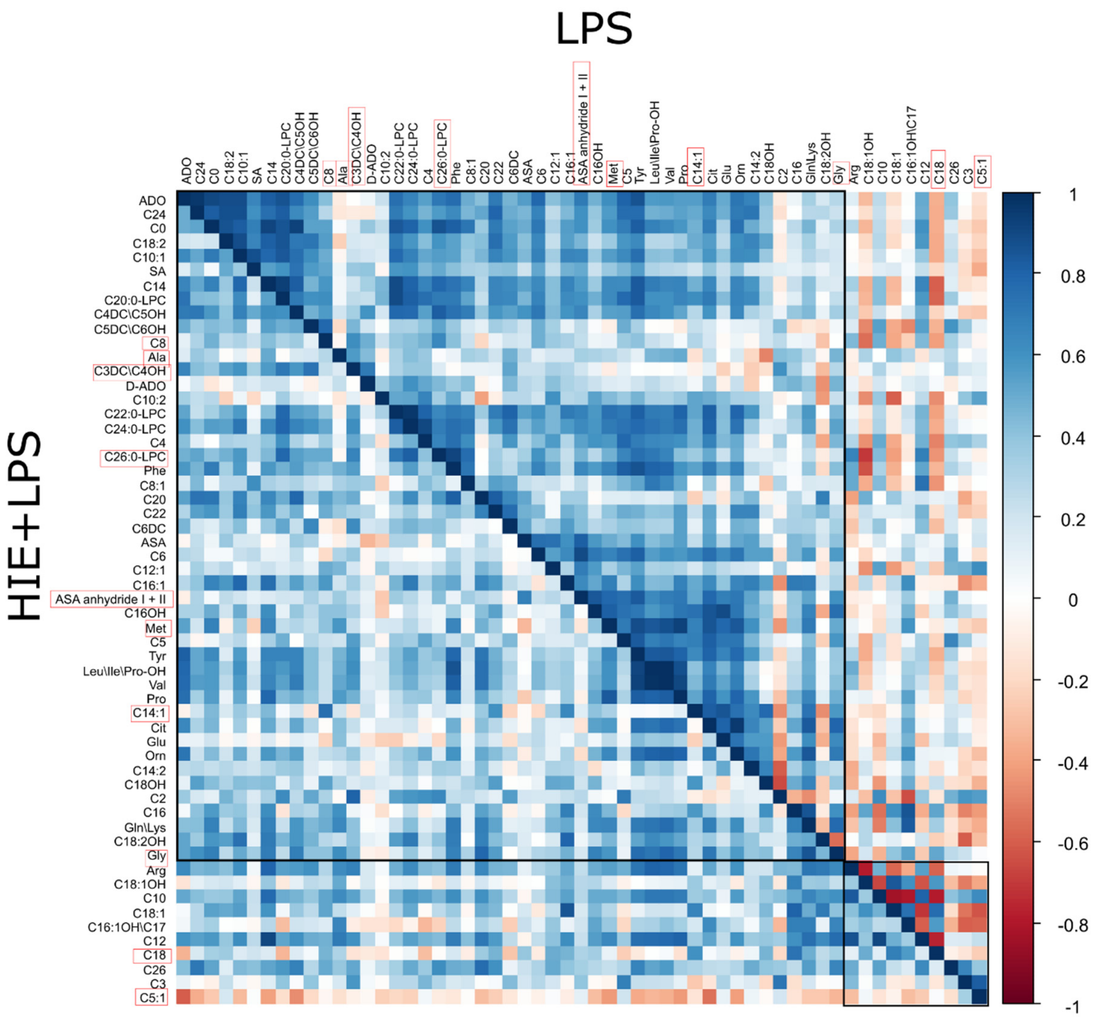


**Figure S2.** Comparison of correlations for the group with inflammation and the group with inflammation and hypoxia/ischemia. Compounds with a statistically significant difference in levels are highlighted in red, clusters of compounds are highlighted in black squares.

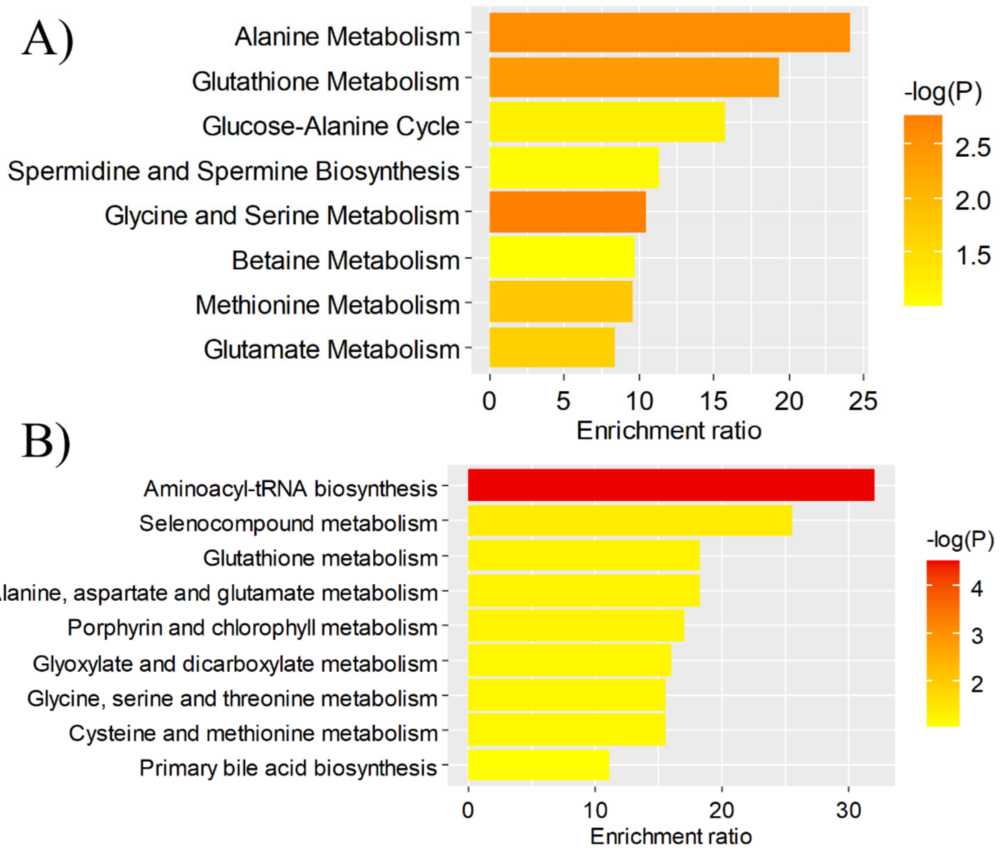


**Figure S3.** Enrichment levels of statistically significantly enriched pathways during ischemia/hypoxia against the inflammation according to **A**) the SMPDB library, **B**) the KEGG library.

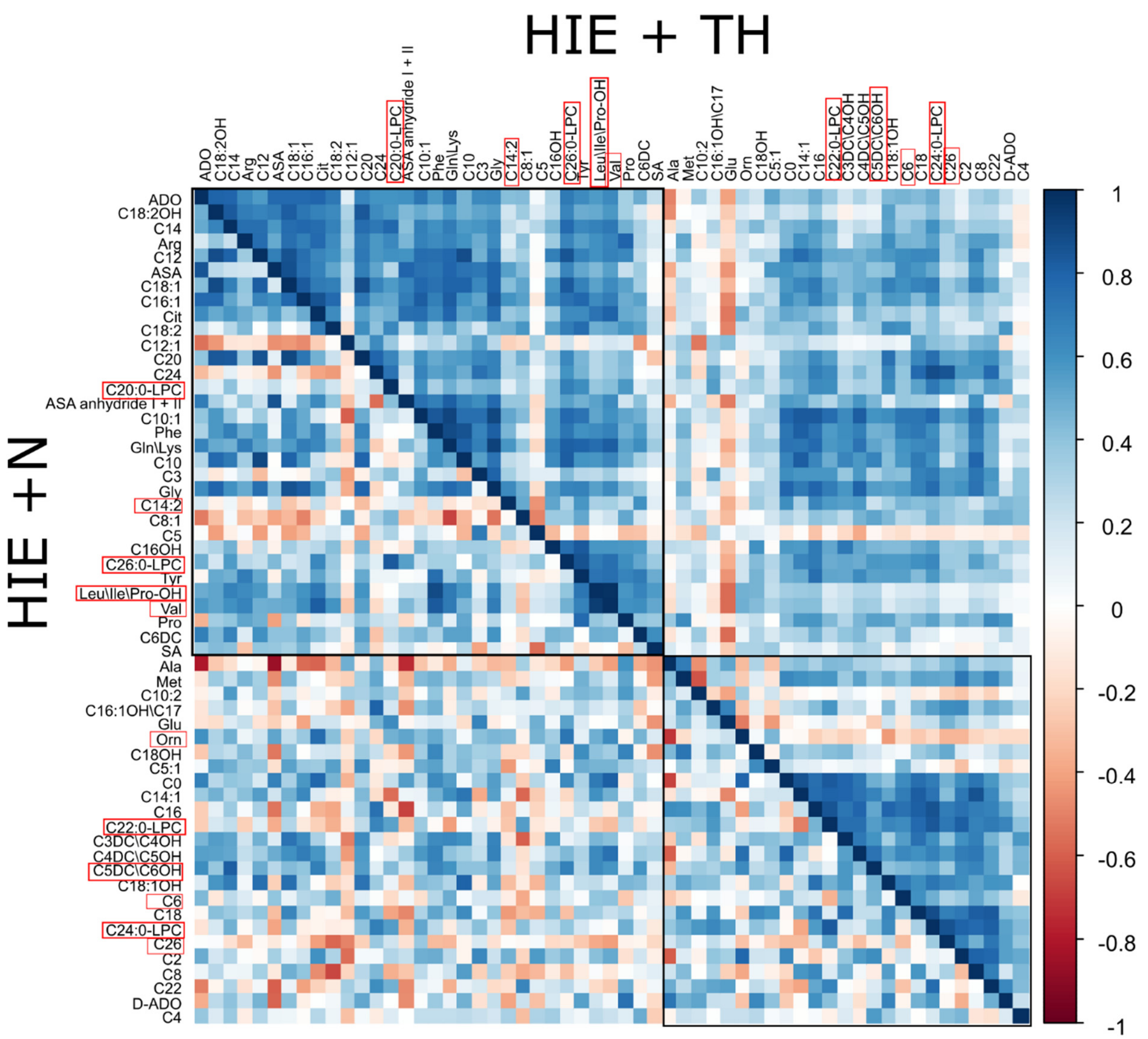


**Figure S4.** Comparison of correlations for the group with normothermia and the group with hypothermia after hypoxia/ischemia. Compounds with a statistically significant difference in levels are highlighted in red, clusters of compounds are highlighted in black squares.

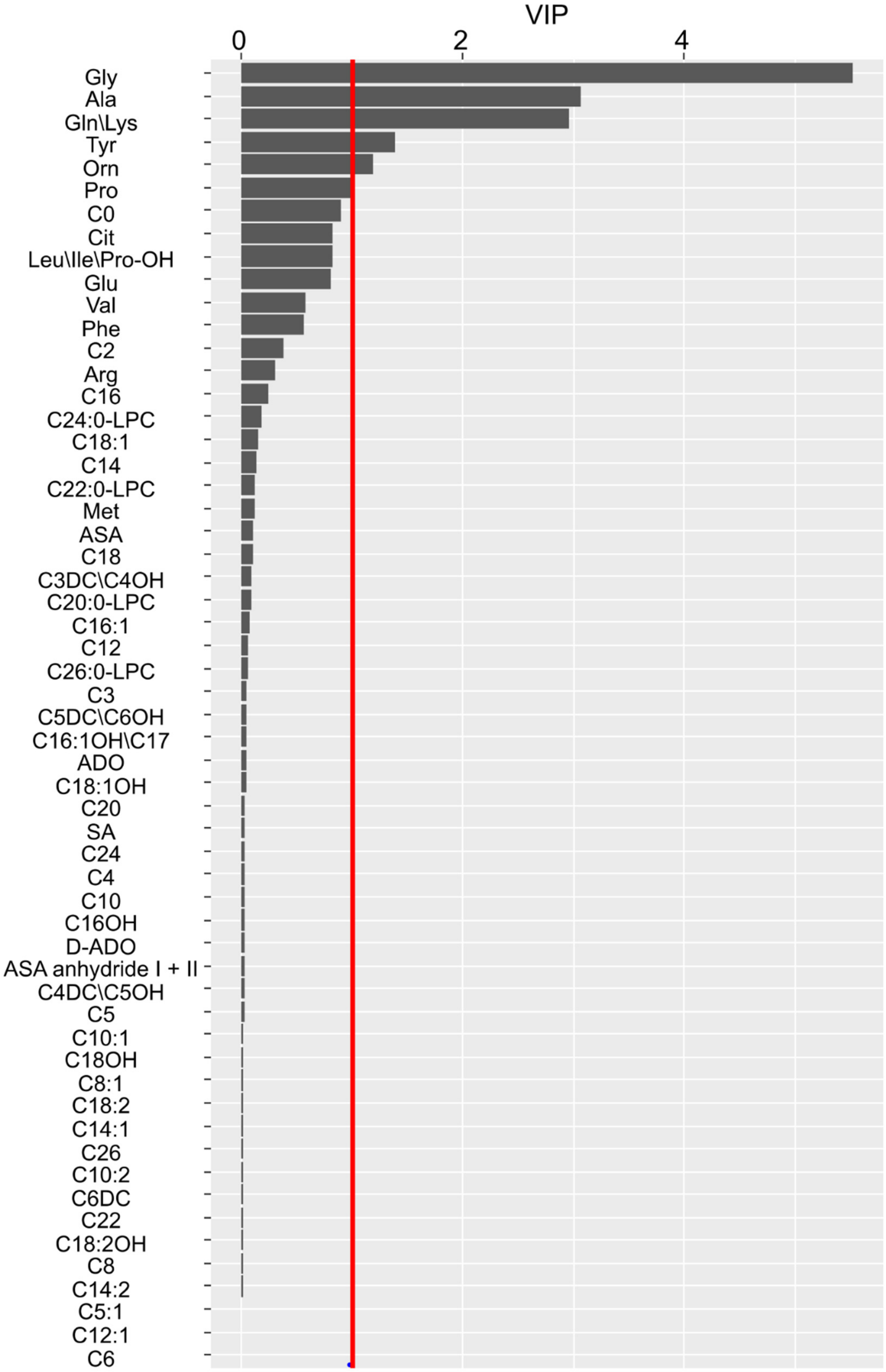


**Figure S5.** Distribution of variable projection values (VIP) in the OPLS-DA model for diagnosing hypoxia using a blood profile three hours after injury. The red line is threshold (VIP = 1) of more important metabolites.

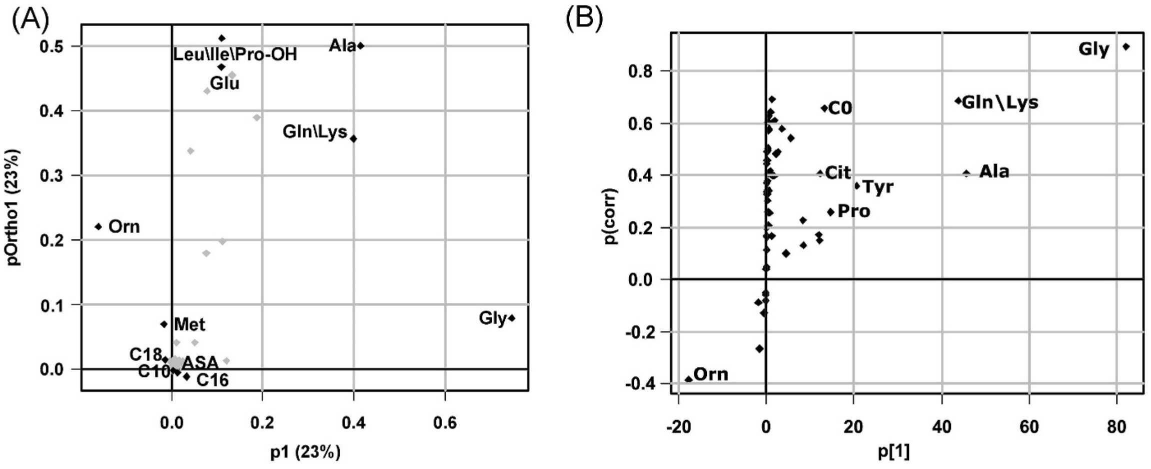


**Figure S6.** Loading plot (**A**) and S-plot (**B**) of compounds, which are included in the OPLS model for hypoxia detection 3 hours after damage.

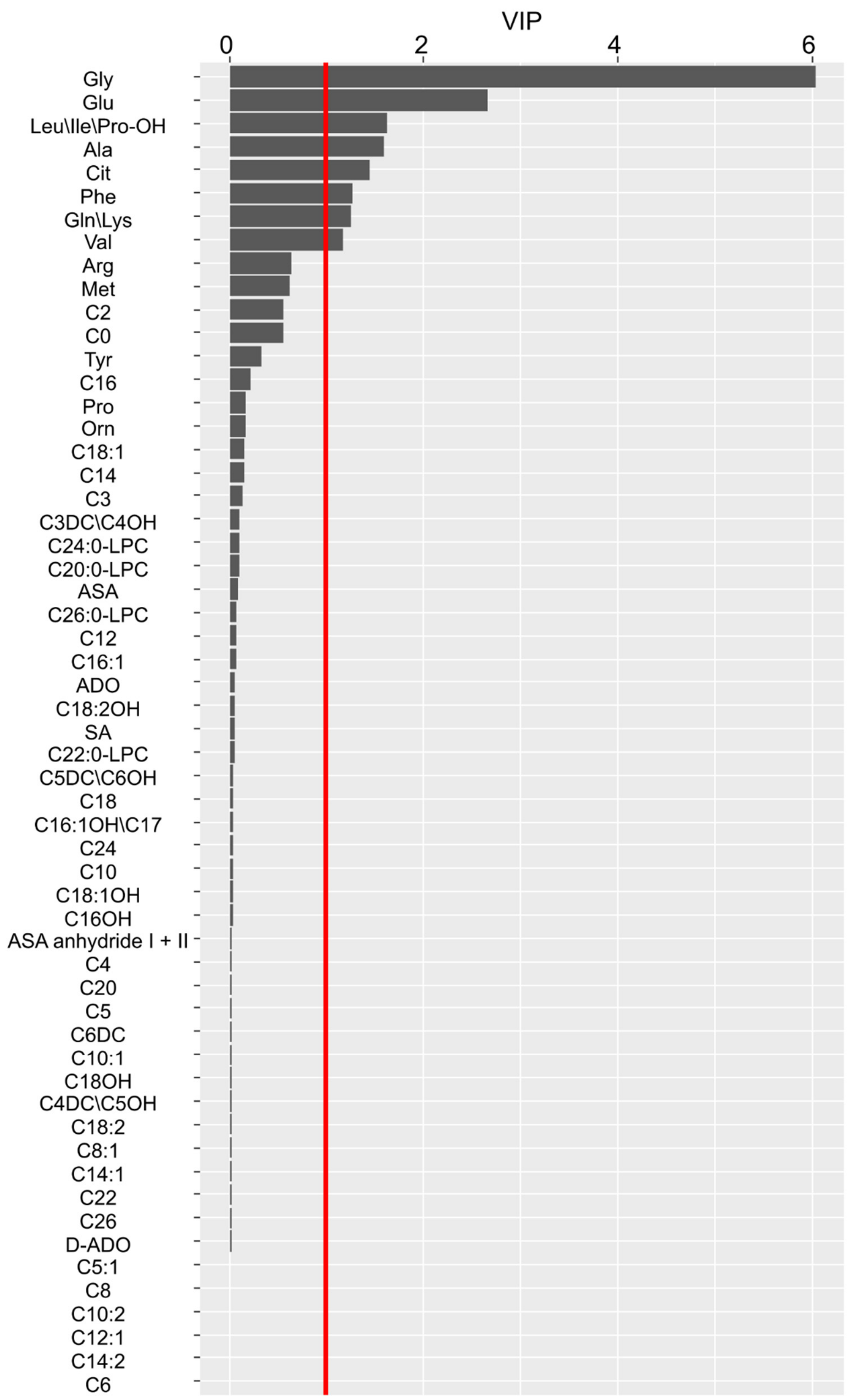


**Figure S7.** Distribution of variable projection values (VIP) in the OPLS-DA model for diagnosing hypoxia using a blood profile six hours after injury. The red line is threshold (VIP = 1) of more important metabolites.

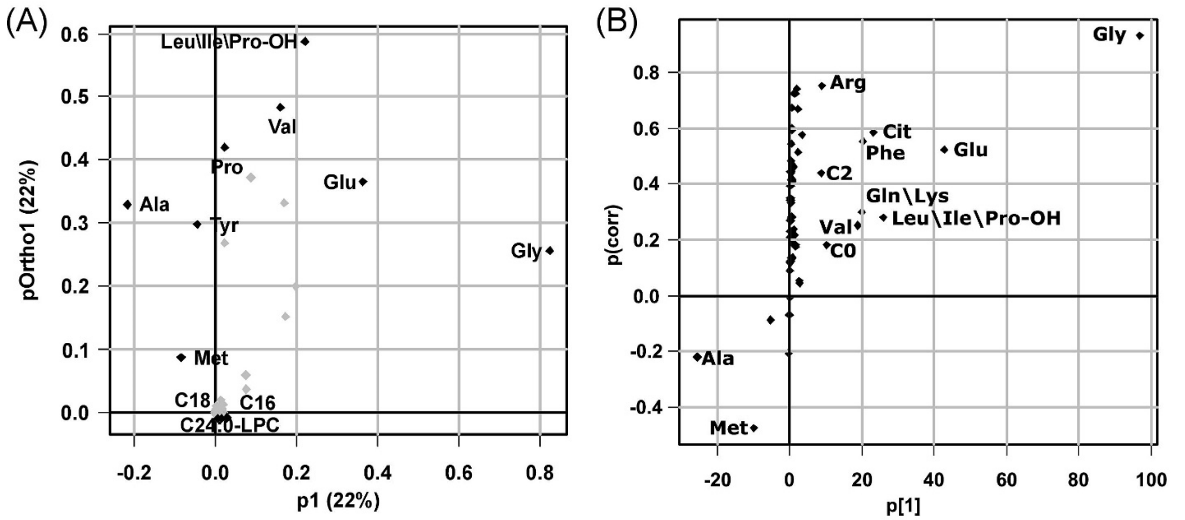


**Figure S8.** Loading plot (**A**) and S-plot (**B**) of compounds, which are included in the OPLS model for hypoxia detection 6 hours after damage.

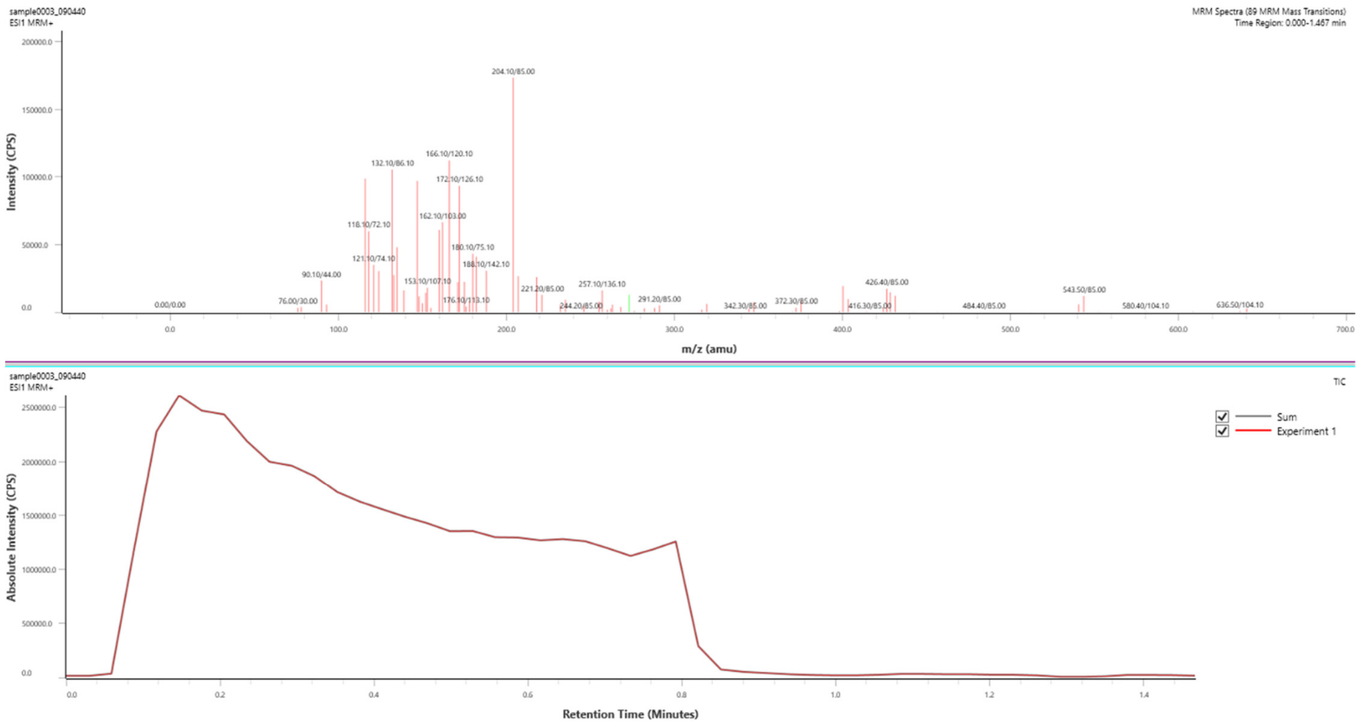


**Figure S9.** Example of a typical flow injection chromatogram obtained for a low quality control (LC) provided by NeoBase™ 2 Non-derivatized MSMS kit on the QSight 225MD tandem mass spectrometer.

**Table S1.** Differences in the molecular profile of blood 3 hours after hypoxia compared to control. Changes with FDR < 0.05 are marked bold.

| Species | control | 3 h | *p* | FDR | FC |
|---|---|---|---|---|---|
| **Gly** | **237(217;279)** | **359(322;384)** | **<0.001** | **0.004** | **1.51** |
| C3DC\C4OH | 0.093(0.082;0.12) | 0.15(0.12;0.16) | 0.003 | 0.08 | 1.61 |
| C5DC\C6OH | 0.022(0.019;0.028) | 0.034(0.026;0.042) | 0.02 | 0.21 | 1.55 |
| C10:2 | 0.002(0.002;0.0028) | 0.004(0.003;0.004) | 0.02 | 0.21 | 2.00 |
| Gln\Lys | 149(126;188) | 202(187;211) | 0.02 | 0.21 | 1.36 |
| C26 | 0.004(0.004;0.005) | 0.005(0.005;0.006) | 0.02 | 0.21 | 1.25 |
| C2 | 3.9(3.2;4.5) | 4.3(4.1;5.6) | 0.03 | 0.21 | 1.10 |
| C18:2 | 0.005(0.0042;0.0068) | 0.009(0.006;0.01) | 0.03 | 0.21 | 1.80 |
| C14 | 0.32(0.28;0.36) | 0.42(0.35;0.44) | 0.04 | 0.27 | 1.31 |
| C24 | 0.034(0.03;0.035) | 0.039(0.035;0.045) | 0.05 | 0.27 | 1.15 |

**Table S2.** Differences in the molecular profile of blood 6 hours after hypoxia compared to control. Changes with FDR < 0.05 are marked bold.

| Species | Control | 6 h | *p* | FDR | FC |
|---|---|---|---|---|---|
| **Gly** | **237(217;279)** | **376(327;448)** | **<0.001** | **0.002** | **1.59** |
| **C26** | **0.004(0.004;0.005)** | **0.0075(0.0058;0.009)** | **<0.001** | **0.009** | **1.88** |
| **Met** | **20(18;22)** | **15(13;17)** | **0.002** | **0.03** | **0.75** |
| **C3DC\C4OH** | **0.093(0.082;0.12)** | **0.16(0.12;0.18)** | **0.002** | **0.03** | **1.72** |
| **C3** | **0.15(0.13;0.16)** | **0.22(0.18;0.28)** | **0.002** | **0.03** | **1.47** |
| **C4** | **0.042(0.04;0.048)** | **0.053(0.05;0.061)** | **0.004** | **0.04** | **1.26** |
| **C18:2** | **0.005(0.0042;0.0068)** | **0.008(0.007;0.009)** | **0.006** | **0.05** | **1.60** |
| C2 | 3.9(3.2;4.5) | 5(4.6;5.8) | 0.009 | 0.06 | 1.28 |
| C5DC\C6OH | 0.022(0.019;0.028) | 0.031(0.029;0.038) | 0.01 | 0.07 | 1.41 |
| C24 | 0.034(0.03;0.035) | 0.040(0.037;0.046) | 0.01 | 0.07 | 1.18 |
| C8:1 | 0.0075(0.0055;0.008) | 0.012(0.0097;0.015) | 0.01 | 0.07 | 1.60 |
| C20 | 0.024(0.022;0.03) | 0.032(0.029;0.039) | 0.02 | 0.08 | 1.33 |
| C5 | 0.03(0.025;0.037) | 0.039(0.036;0.045) | 0.02 | 0.11 | 1.30 |
| SA | 0.33(0.32;0.36) | 0.37(0.35;0.38) | 0.03 | 0.11 | 1.12 |
| C10:2 | 0.002(0.002;0.0028) | 0.0035(0.0028;0.0052) | 0.03 | 0.13 | 1.75 |

**Table S3.** Pathways enriched with markers for a three-hour time period after ischemia/hypoxia relative to the control using the SMPDB and KEGG libraries, the number of compounds in them, the degree of enrichment, the number of markers in them, the probability of a random hit.

| Library | Pathway | Total | Enrichment, Mean | Hits, Median | *p*, Mean |
|---|---|---|---|---|---|
| SMPDB | Ammonia Recycling | 32 | 15.99 | 2 | 0.03 |
| SMPDB | Beta Oxidation of Very Long Chain Fatty Acids | 17 | 18.41 | 1 | 0.05 |
| SMPDB | Alanine Metabolism | 17 | 18.41 | 1 | 0.05 |
| SMPDB | Carnitine Synthesis | 22 | 23.26 | 2 | 0.02 |
| KEGG | Glyoxylate and dicarboxylate metabolism | 32 | 34.65 | 2 | 0.01 |
| KEGG | Aminoacyl-tRNA biosynthesis | 48 | 31.99 | 2 | 0.001 |
| KEGG | Glutathione metabolism | 28 | 24.36 | 1 | 0.04 |
| KEGG | Porphyrin and chlorophyll metabolism | 30 | 22.74 | 1 | 0.04 |
| KEGG | Glycine, serine and threonine metabolism | 33 | 20.67 | 1 | 0.049 |

**Table S4.** Pathways enriched with markers for a six-hour time period after ischemia/hypoxia relative to the control using the SMPDB and KEGG libraries, the number of compounds in them, the degree of enrichment, the number of markers in them, the probability of a random hit.

| Library | Pathway | Total | Enrichment, Mean | Hits, Median | *p*, Mean |
|---|---|---|---|---|---|
| SMPDB | Oxidation of Branched Chain Fatty Acids | 26 | 19.61 | 2 | 0.004 |
| SMPDB | Methionine Metabolism | 43 | 11.90 | 2 | 0.01 |
| SMPDB | Glycine and Serine Metabolism | 59 | 8.70 | 2 | 0.02 |
| KEGG | Aminoacyl-tRNA biosynthesis | 48 | 32.00 | 2 | <0.001 |
| KEGG | Glutathione metabolism | 28 | 27.40 | 1 | 0.04 |
| KEGG | Porphyrin and chlorophyll metabolism | 30 | 25.58 | 1 | 0.04 |
| KEGG | Glyoxylate and dicarboxylate metabolism | 32 | 23.98 | 1 | 0.04 |
| KEGG | Glycine, serine and threonine metabolism | 33 | 23.26 | 1 | 0.04 |
| KEGG | Cysteine and methionine metabolism | 33 | 23.26 | 1 | 0.04 |

Table S5. Differences in the molecular profile of blood after hypothermic regimen compared to normothermic regimen in a rat model of hypoxic-ischemic injury.

| Compounds | Normotermia | Hypotermia | *p* | FDR | FC |
|---|---|---|---|---|---|
| C22:0-LPC | 1.5(1.3;1.7) | 1.2(1.1;1.4) | 0.006 | 0.20 | 0.80 |
| C24:0-LPC | 2.3(2.1;2.7) | 1.9(1.8;2.2) | 0.007 | 0.20 | 0.83 |
| Orn | 17(13;21) | 25(20;41) | 0.01 | 0.23 | 1.47 |
| C26 | 0.007(0.0062;0.008) | 0.006(0.005;0.007) | 0.02 | 0.25 | 0.86 |
| Val | 110(104;124) | 146(120;171) | 0.02 | 0.25 | 1.33 |
| C20:0-LPC | 2.8(1.9;3.2) | 2.1(1.7;2.5) | 0.03 | 0.25 | 0.75 |
| C26:0-LPC | 0.49(0.42;0.52) | 0.39(0.36;0.44) | 0.04 | 0.25 | 0.80 |
| C5DC\C6OH | 0.043(0.034;0.048) | 0.029(0.027;0.041) | 0.04 | 0.25 | 0.67 |
| Leu\Ile\Pro-OH | 160(154;188) | 209(177;229) | 0.04 | 0.25 | 1.31 |
| C6 | 0.004(0.003;0.005) | 0.003(0.002;0.0032) | 0.04 | 0.25 | 0.75 |
| C14:2 | 0.007(0.0062;0.0087) | 0.006(0.005;0.007) | 0.05 | 0.25 | 0.86 |

**Table S6.** Parameters of the logistic regression model for the diagnosis of ischemia/hypoxia after 3 hours. Coefficient β, confidence interval CI β, Wald criteria Z, coefficient zero-probability P are provided.

| Variable | β | CI β | Z | *p* |
|---|---|---|---|---|
| Intercept | -6.64 | -14.06 - -2.52 | -2.43 | 0.02 |
| Glycine^2 | $7.37*10^{-5}$ | $3.01*10^{-5}$ - $1.56*10^{-4}$ | 2.49 | 0.01 |

**Table S7.** Parameters of the logistic regression model for the diagnosis of ischemia/hypoxia after 6 hours. Coefficient β, confidence interval CI β, Wald criteria Z, coefficient zero-probability P are provided

| Variable | β | CI β | Z | *p* |
|---|---|---|---|---|
| Intercept | -9.13 | -23.50 - -3.14 | -1.96 | 0.05 |
| Glycine^2 | $9.54*10^{-5}$ | $3.36*10^{-5}$ - $2.43*10^{-4}$ | 1.98 | 0.048 |

**Table S8.** MS parameters used for FIA-MS/MS analysis by NeoBase™ 2 Non-derivatized MSMS kit. For each compound, MRM transition (Q1 and Q3), entrance voltage, collision cell lens 2, collision energy, and dwell time are shown. IS - internal standard.

| Compound | Q1 | Q3 | Entrance Voltage | Dwell Time | Collision Cell Lens 2 | Collision Energy |
|---|---|---|---|---|---|---|
| Gly | 76 | 30 | 30 | 15 | -100 | -50 |
| Gly IS | 78 | 32 | 30 | 15 | -100 | -50 |
| Ala | 90,1 | 44 | 30 | 15 | -100 | -50 |
| Ala IS | 93,1 | 47,1 | 30 | 15 | -100 | -50 |
| Pro | 116,1 | 70,1 | 30 | 15 | -100 | -50 |
| Pro IS | 121,1 | 74,1 | 30 | 15 | -100 | -50 |
| Val | 118,1 | 72,1 | 30 | 15 | -100 | -50 |
| Val IS | 124,1 | 77,1 | 30 | 15 | -100 | -50 |
| Leu\Ile\Pro-OH | 132,1 | 86,1 | 30 | 15 | -100 | -50 |
| Leu\Ile\Pro-OH IS | 135,1 | 89,1 | 30 | 15 | -100 | -50 |
| Orn | 133,1 | 70,1 | 30 | 15 | -100 | -50 |
| Orn IS | 139,1 | 76,1 | 30 | 15 | -100 | -50 |
| Gln\Lys | 147,1 | 84 | 30 | 15 | -100 | -50 |
| Glu | 148,1 | 84 | 30 | 15 | -100 | -50 |
| Gln\Lys IS | 152,1 | 88,1 | 30 | 15 | -100 | -50 |
| Met | 150,1 | 104,1 | 30 | 15 | -100 | -50 |
| Met IS | 153,1 | 107,1 | 30 | 15 | -100 | -50 |
| SA | 155,1 | 109,1 | 30 | 15 | -100 | -50 |
| SA IS | 160,1 | 114,1 | 30 | 15 | -100 | -50 |
| Phe | 166,1 | 120,1 | 30 | 15 | -100 | -50 |
| Phe IS | 172,1 | 126,1 | 30 | 15 | -100 | -50 |
| Arg | 175,1 | 70,1 | 30 | 15 | -100 | -50 |
| Arg IS | 180,1 | 75,1 | 30 | 15 | -100 | -50 |
| Cit | 176,1 | 113,1 | 30 | 15 | -100 | -50 |
| Cit IS | 178,1 | 115,1 | 30 | 15 | -100 | -50 |
| Tyr | 182,1 | 136,1 | 30 | 15 | -100 | -50 |
| Tyr IS | 188,1 | 142,1 | 30 | 15 | -100 | -50 |
| D-ADO | 252,1 | 136,1 | 30 | 15 | -100 | -50 |
| D-ADO IS | 257,1 | 136,1 | 30 | 15 | -100 | -50 |
| ADO | 268,1 | 136,1 | 30 | 15 | -100 | -50 |
| ADO IS | 273,1 | 136,1 | 30 | 15 | -100 | -50 |
| ASA anhydride I + II | 273,1 | 70,1 | 30 | 15 | -100 | -50 |
| ASA | 291,1 | 70,1 | 30 | 15 | -100 | -50 |
| C0 | 162,1 | 103 | 30 | 15 | -100 | -50 |
| C0 IS | 171,2 | 103 | 30 | 15 | -100 | -50 |
| C2 | 204,1 | 85 | 30 | 15 | -100 | -50 |
| C2 IS | 207,1 | 85 | 30 | 15 | -100 | -50 |
| C3 | 218,1 | 85 | 30 | 15 | -100 | -50 |
| C3 IS | 221,2 | 85 | 30 | 15 | -100 | -50 |
| C3DC\C4OH | 248,1 | 85 | 30 | 15 | -100 | -50 |
| C4 | 232,2 | 85 | 30 | 15 | -100 | -50 |
| C4 IS | 235,2 | 85 | 30 | 15 | -100 | -50 |

| | | | | | | |
|---|---|---|---|---|---|---|
| C5:1 | 244,2 | 85 | 30 | 15 | -100 | -50 |
| C5 | 246,2 | 85 | 30 | 15 | -100 | -50 |
| C4DC\C5OH | 262,2 | 85 | 30 | 15 | -100 | -50 |
| C5 IS | 255,2 | 85 | 30 | 15 | -100 | -50 |
| C6 | 260,2 | 85 | 30 | 15 | -100 | -50 |
| C6 IS | 263,2 | 85 | 30 | 15 | -100 | -50 |
| C5DC\C6OH | 276,2 | 85 | 30 | 15 | -100 | -50 |
| C6DC | 290,2 | 85 | 30 | 15 | -100 | -50 |
| C5DC\C6OH IS | 282,2 | 85 | 30 | 15 | -100 | -50 |
| C8:1 | 286,2 | 85 | 30 | 15 | -100 | -50 |
| C8 | 288,2 | 85 | 30 | 15 | -100 | -50 |
| C8 IS | 291,2 | 85 | 30 | 15 | -100 | -50 |
| C10:2 | 312,2 | 85 | 30 | 15 | -100 | -50 |
| C10:1 | 314,2 | 85 | 30 | 15 | -100 | -50 |
| C10 | 316,2 | 85 | 30 | 15 | -100 | -50 |
| C10 IS | 319,3 | 85 | 30 | 15 | -100 | -50 |
| C12:1 | 342,3 | 85 | 30 | 15 | -100 | -50 |
| C12 | 344,3 | 85 | 30 | 15 | -100 | -50 |
| C12 IS | 347,3 | 85 | 30 | 15 | -100 | -50 |
| C14:2 | 368,3 | 85 | 30 | 15 | -100 | -50 |
| C14:1 | 370,3 | 85 | 30 | 15 | -100 | -50 |
| C14 | 372,3 | 85 | 30 | 15 | -100 | -50 |
| C14OH | 388,3 | 85 | 30 | 15 | -100 | -50 |
| C14 IS | 375,3 | 85 | 30 | 15 | -100 | -50 |
| C16:1 | 398,3 | 85 | 30 | 15 | -100 | -50 |
| C16 | 400,3 | 85 | 30 | 15 | -100 | -50 |
| C16:1OH\C17 | 414,3 | 85 | 30 | 15 | -100 | -50 |
| C16OH | 416,3 | 85 | 30 | 15 | -100 | -50 |
| C16 IS | 403,4 | 85 | 30 | 15 | -100 | -50 |
| C18:2 | 424,3 | 85 | 30 | 15 | -100 | -50 |
| C18:1 | 426,4 | 85 | 30 | 15 | -100 | -50 |
| C18 | 428,4 | 85 | 30 | 15 | -100 | -50 |
| C18:2OH | 440,3 | 85 | 30 | 15 | -100 | -50 |
| C18:1OH | 442,4 | 85 | 30 | 15 | -100 | -50 |
| C18OH | 444,4 | 85 | 30 | 15 | -100 | -50 |
| C18 IS | 431,4 | 85 | 30 | 15 | -100 | -50 |
| C20 | 456,4 | 85 | 30 | 15 | -100 | -50 |
| C22 | 484,4 | 85 | 30 | 15 | -100 | -50 |
| C24 | 512,5 | 85 | 30 | 15 | -100 | -50 |
| C26 | 540,5 | 85 | 30 | 15 | -100 | -50 |
| C26 IS | 543,5 | 85 | 30 | 15 | -100 | -50 |
| C20:0-LPC | 552,4 | 104,1 | 30 | 15 | -100 | -50 |
| C22:0-LPC | 580,4 | 104,1 | 30 | 15 | -100 | -50 |
| C24:0-LPC | 608,5 | 104,1 | 30 | 15 | -100 | -50 |
| C26:0-LPC | 636,5 | 104,1 | 30 | 15 | -100 | -50 |
| C26:0-LPC IS | 640,5 | 104,1 | 30 | 15 | -100 | -50 |

**Table S9.** Results of mass-spectrometry analysis, include information about MRM transition, retention time, preprocession parametr, peak height and area, concentration in fmol/mkl, for serum metabolites and internal standards in study sample, quality control sample, low concentration sample and hight concentration sample. Containing in individual excel file.
