# Supplementary material for: Metabolite Biomarkers for Early Ischemic–Hypoxic Encephalopathy: An Experimental Study Using the NeoBase 2 MSMS Kit in a Rat Model
Source: Int J Mol Sci. 2024 Feb 7;25(4):2035. doi: 10.3390/ijms25042035 (PMC10888647; doi:10.3390/ijms25042035)
Supplement: Supplementary file 1 [file ijms-25-02035-s001.zip › ijms-2782235-supplementary/Titles of supplementary tables.pdf]

Table S1. Differences in the molecular profile of blood 3 hours after hypoxia compared to control. Changes with FDR < 0.05 are marked bold.

Table S2. Differences in the molecular profile of blood 6 hours after hypoxia compared to control. Changes with FDR < 0.05 are marked bold.

Table S3. Pathways enriched with markers for a three-hour time period after ischemia/hypoxia relative to the control using the SMPDB and KEGG libraries, the number of compounds in them, the degree of enrichment, the number of markers in them, the probability of a random hit.

Table S4. Pathways enriched with markers for a six-hour time period after ischemia/hypoxia relative to the control using the SMPDB and KEGG libraries, the number of compounds in them, the degree of enrichment, the number of markers in them, the probability of a random hit.

Table S5. Differences in the molecular profile of blood after hypothermic regimen compared to normothermic regimen in a rat model of hypoxic-ischemic injury.

Table S6. Parameters of the logistic regression model for the diagnosis of ischemia/hypoxia after 3 hours. Coefficient β, confidence interval CI β, Wald criteria Z, coefficient zero-probability P are provided.

Table S7. Parameters of the logistic regression model for the diagnosis of ischemia/hypoxia after 6 hours. Coefficient β, confidence interval CI β, Wald criteria Z, coefficient zero-probability P are provided

Table S8. MS parameters used for FIA-MS/MS analysis by NeoBase™ 2 Non-derivatized MSMS kit. For each compound, MRM transition (Q1 and Q3), entrance voltage, collision cell lens 2, collision energy, and dwell time are shown. IS - internal standard.

Table S9. Results of mass-spectrometry analysis, include information about MRM transition, retention time, preprocession parametr, peak height and area, concentration in fmol/mkl, for serum metabolites and internal standards in study sample, quality control sample, low concentration sample and hight concentration sample. Containing in individual file.
